# Sensorineural hearing loss in early childhood after passing newborn hearing screening

**DOI:** 10.1007/s00405-026-10435-1

**Published:** 2026-07-19

**Authors:** I. E. C. Smetsers, N. Uilenburg, R. J. E. Pennings, C. P. Lanting, F. L. J. Cals

**Affiliations:** 1 Department of Otorhinolaryngology and Head and Neck Surgery, Radboudumc, Geert Grooteplein Zuid 10, Nijmegen, The Netherlands; 2 Dutch Foundation for the Deaf and Hard of Hearing Child, Amsterdam, Netherlands

**Keywords:** Newborn hearing screening, Pass screening, Otoacoustic emissions, Automated auditory brainstem response, Sensorineural hearing loss

## Abstract

**Introduction:**

The newborn hearing screening (NHS) program in the Netherlands consist of two rounds of otoacoustic emissions (OAE) and a third round of automated auditory brainstem responses (AABR). The program identifies about 200 newborns with sensorineural hearing loss (SNHL) annually, enabling early intervention. Nevertheless, a subgroup of patients passes the NHS but present with SNHL in early childhood.

**Purpose:**

To determine the number of patients diagnosed with SNHL (< 5 years) who initially passed their NHS**,** and characterize this group in terms of patient demographics, NHS results, hearing loss (HL) characteristics and etiology of, to identify opportunities for earlier detection.

**Methods:**

A retrospective descriptive study was conducted on patients (< 5 years) seen between 2014 and 2024, passed their NHS and had SNHL with an unknown cause, for which they underwent etiological diagnostics.

**Results:**

119 patients with 205 ears were included. Three-fourth of patients had an abnormal speech and language development (SLD). A relatively large number of ears passed in the third AABR round (36%). HL characteristics were highly variable. An etiological diagnosis was found in 74 patients (62%), most commonly a genetic cause (40%) or a cochleovestibular malformation (18%).

**Conclusions:**

We estimate that in the Netherlands approximately 50 patients per year present with SNHL (< 5 years) after passing their NHS, of which the majority manifest abnormal SLD. Highly variable HL characteristics and etiology make it difficult to identify them. Nevertheless, a possibility for earlier detection is a second screening for specific subgroups. No evidence of false negatives during NHS was found.

## Introduction

Congenital hearing loss affects approximately 1–2 per 1,000 newborns [1, 2]. It has been shown that early detection of permanent hearing loss (HL) is critical, as timely intervention improves speech and language development (SLD), social development, as well as overall quality of life [3–6]. To facilitate early intervention, newborn hearing screening (NHS) programs have been developed, aiming to detect HL greater than 35–40 dB in infants before the age of three months [7, 8]. These programs have been proven to be cost-effective [5, 9].

Screening protocols of NHS differ internationally. A two-step Automated Auditory Brainstem Response (AABR) NHS program was introduced in 1998 for neonatal intensive care unit (NICU) newborns in the Netherlands. This was followed by a nationwide universal NHS for (near) term newborns in 2006, which consists of a three-round screening program performed by youth healthcare organizations [7, 8]. In the initial and second round an otoacoustic emissions (OAE) test is performed, in which a click stimulus between 1000-4000 Hz is used to generate OAE, which are typically absent in HL greater than 40-50 dB. [1, 10] In the third round an AABR test is performed, in which a composed click stimulus of 35 dB at 750-5000 Hz is administered and the response to the stimulus in the vestibulocochlear nerve and brainstem is measured [1, 11]. Newborns who pass a test receive no further screening. If all three tests result in a refer, the infant is referred for diagnostic evaluation of their hearing [1, 7].

**Fig. 1:**
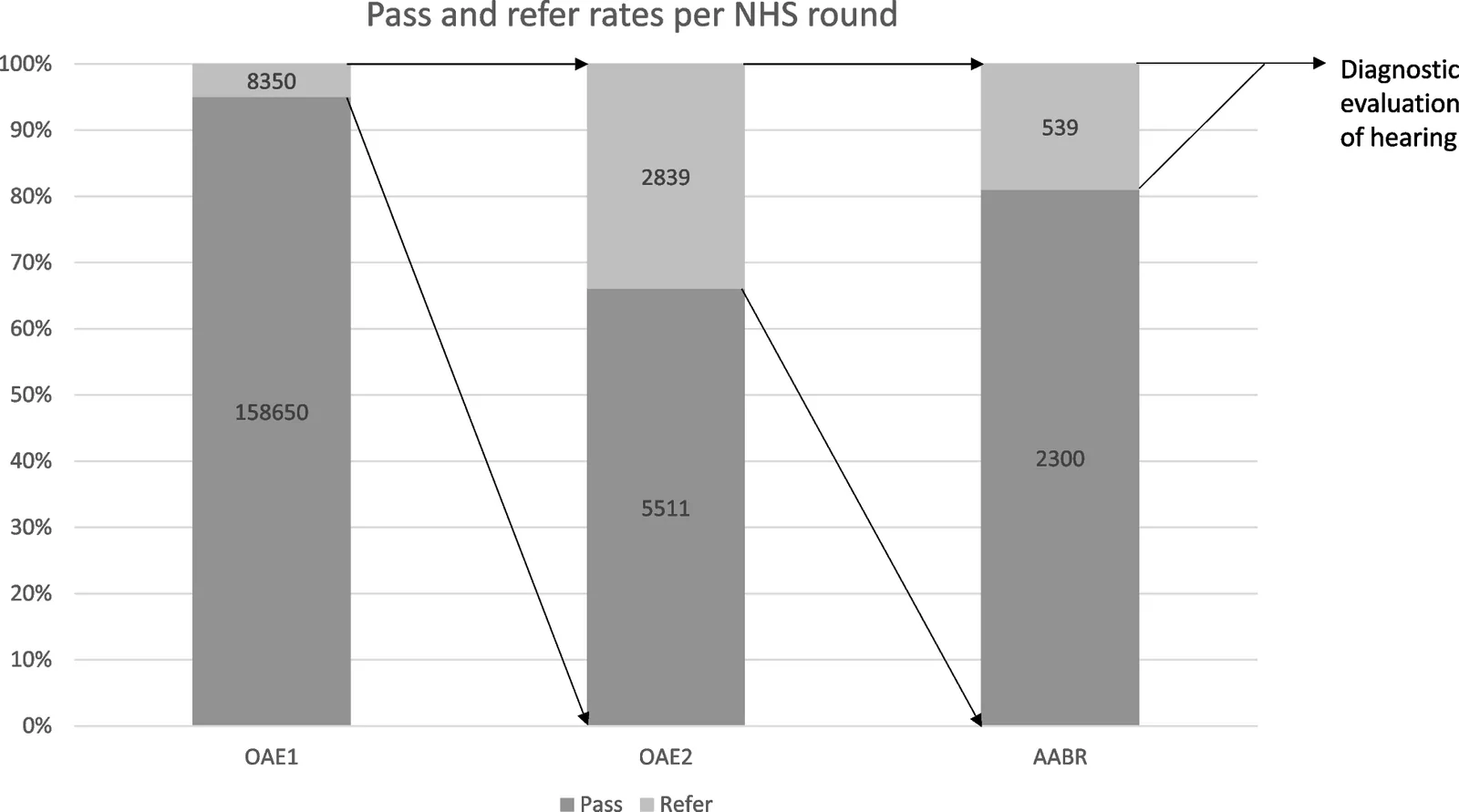
Mean pass and refer rates (2006–2023) per NHS round with number of participants per round (total 167,000) [7, 8]

Since the implementation in 2006 participation rates of the Dutch NHS have consistently exceeded 98%. On average, approximately 167,000 infants are screened annually. The mean rates of ‘refer’ after the first, second and third round are 5%, 34%, and 19%, respectively (Fig. 1) [7, 8]. Of the 500–600 infants referred for diagnostic evaluation, approximately 200 infants are diagnosed with sensorineural hearing loss (SNHL) each year, of which approximately 60% concerns bilateral SNHL and 40% unilateral SNHL. This is equivalent to the prevalence of congenital SNHL (1.2 per 1000) [1, 2].

Nevertheless, a subset of patients passes the NHS but present with SNHL in early childhood. For example, in a study by Korver et al. on children with permanent hearing impairment at age three to five years who received NHS, HL was not detected with NHS in 15 out of 80 children (18.7%), but afterwards [6]. In other literature, the described prevalence of permanent HL after passing NHS in children under 12 years varies between 0.32 and 77.87 per 1,000 [12, 13]. Despite this high variability, the research highlights that it concerns a substantial number of patients. Clear information on this population remains limited. Given the well-established association between delayed detection of HL and adverse developmental outcomes, it is critical to characterize this subgroup to identify opportunities for earlier detection.

Therefore, this study aims to determine the number of patients diagnosed with SNHL before the age of five, who initially passed their NHS. In addition, this study seeks to characterize this group in terms of patient demographics, NHS results, HL characteristics and etiology, to define possible modifications to the screening protocol to facilitate earlier detection.

## Methods

### Data acquisition

This retrospective descriptive study was conducted at the Department of Otorhinolaryngology of the Radboud University Medical Center in Nijmegen, the Netherlands, using electronic medical records of patients seen in our outpatient clinic between 2014 and 2024. The study was evaluated as not subject to the Medical Research Involving Human Subjects Act by the Medical Ethics Review Committee East Netherlands (file number 2025–18442).

All patients with SNHL or mixed HL with an unknown cause under the age of five years were considered for inclusion. Inclusion required either a bilateral pass on the NHS, or a unilateral pass in children who were later diagnosed with bilateral SNHL. In addition, all patients had undergone at least one form of etiological diagnostics. Patients who followed the alternative NICU protocol (i.e. two round of AABR) were excluded.

Patient records were reviewed for the following outcome variables:

1. **Demographics:** Gender and age at referral. Since all patients were referred for etiological diagnostics and not diagnostics of hearing itself, the age of onset of HL could not reliably be determined. Therefore, the age at referral was used for further analysis.
2. **NHS results:** After parental informed consent the official NHS results were retrieved from the National Institute for Public Health and the Environment (RIVM). Results included a ‘pass’ or ‘refer’ for each ear per screening round.
3. **Hearing loss:** HL was analyzed per ear. Ears with normal hearing, conductive HL or a refer on NHS were excluded. Audiometry (pure tone audiograms (*n* = 113) or brainstem evoked response audiometry (*n* = 6)) obtained at presentation was evaluated using the ERN-CRANIO rare genetic deafness criteria, based on recommendations by the GENDEAF study group [14]. Severity of HL (normal 0-20 dB; mild 20-40 dB; moderate 41-70 dB; severe 41-90 dB; profound > 91 dB) was classified using the pure tone average (PTA) of thresholds at 1, 2 and 4 kHz. For low-frequency HL the PTA of 0.5, 1 and 2 kHz was used instead. When available, follow-up audiometry was used to evaluate progression. Progression of HL was evaluated over a ten-year period. An increase in HL threshold (PTA) of < 15 dB was defined as no progression. A ≥ 15 dB increase in HL threshold, even in a period shorter than ten years, was considered as progression. Audiogram configurations included:
  - Low-frequency (> 15 dB HL difference from the poorer low frequency thresholds to the higher frequencies)
  - Mid-frequency (> 15 dB HL difference between poorest thresholds in the mid-frequencies compared to those at higher and lower frequencies)
  - High-frequency gradual (15–29 dB HL difference between PTA 0.5, 1 kHz and PTA 4, 8 kHz)
  - High-frequency steep (> 30 dB difference between PTA 0.5, 1 kHz and PTA 4, 8 kHz)
  - Flat (< 15 dB difference between PTA 0.25, 0.5 kHz and PTA 1, 2 kHz and PTA 4, 8 kHz)
4. **Etiological diagnostics of HL:** Genetic testing (targeted single gene test or Whole Exome Sequencing with a SNHL-panel), inner ear imaging (computed tomography (CT) and/or magnetic resonance imaging (MRI)) and/or analysis for congenital cytomegalovirus infection (cCMV) in the newborn blood spot test. The etiology of HL was based on interpretation of diagnostic findings. For genetic etiologies, only patients with class 4 (likely pathogenic) or class 5 variants (pathogenic) were consisted causative [15].
5. **Additional clinical data:** Data on speech language development (SLD), family history of SNHL, and risk factors (asphyxia, hyperbilirubinemia, recurrent otitis, ototoxic medication, meningitis, head trauma, admittance to NICU, and noise exposure) were retrieved from electronic patients’ files.

### Statistical analysis

Continuous data were tested for normality using the Shapiro–Wilk test. Because data was non-normally distributed, the Mann–Whitney U test was used for ordinal data and Chi-square test or Fisher’s exact test were performed on categorical data. After univariate analysis, factors were selected for multivariable analysis and entered into a logistic regression model. A *p*-value of < 0.05 was chosen as a threshold of significance. IBM SPSS Statistics was used for Windows, Version 24.0 (IBM Corp., Armonk, NY) for statistical analyses.

## Results

### Population

Between 2014 and 2024, in total 119 patients (238 ears) under the age of five years passed their NHS while presenting with SNHL or mixed HL with an unknown cause, for which they received etiological diagnostics. After reviewing patient files, 33 ears were excluded because of a unilateral refer on the NHS in that ear, or only unilateral (SN)HL being present. This resulted in 119 patients and 205 ears being included this study.

### Demographics

The study population consisted of an equal distribution of male and female patients (Table 1). The mean age at referral was 3.4 years (range: 0.0–5.0 years), with the majority (73%) being referred after the age of three years. It is noteworthy that eight out of nine patients who were referred before the age of one had a unilateral refer on the NHS. It is likely that contralateral HL was detected at an early stage, as they were referred for further diagnostic evaluation due to referral on the NHS. No significant differences were found when comparing the type of passed screening test (i.e. OAE or AABR) between different age groups (*p* = 0.208).

**Table 1:** Characteristics per patient

		N_patients_ = 119 N (%)
Gender	Male Female	60 (50.4) 59 (49.6)
Age at referral	years 1–2 years 2–3 years 3–4 years 4–5 years	9 (7.6) 5 (4.2) 18 (15.1) 39 (32.8) 48 (40.3)
Pass NHS	Bilateral Unilateral Missing data	92 (86.8) 14 (13.2) 13
Hearing loss	Bilateral symmetrical Bilateral asymmetrical Unilateral	78 (65.6) 23 (19.3) 18 (15.1)
Speech and language development	Normal Abnormal - Delayed - Stagnated - No development Missing data	27 (24.8) 82 (75.2) 74 (90.2) 6 (7.3) 2 (2.4) 10
Speech therapy	Yes No Missing data	73 (70.2) 31 (29.8) 15
Special education	Yes No	4 (3.4) 115 (96.6)

### NHS results

Official NHS results were retrieved for 106 patients. In the remaining 13 patients, NHS results could not be retrieved, due to absent informed consent or because the NHS was conducted in another country. According to our electronic patient records, all 13 patients passed the NHS for both ears. However, they were excluded from further analysis since this could not be confirmed.

Among the 106 patients with official NHS results, 92 patients (87%) passed bilaterally, and 14 patients (13%) passed unilaterally but presented with bilateral HL (Table 1). Regarding the different screening stages, 105 ears (59%) passed in the first stage using OAE, 10 ears (6%) passed in the second round with OAE, and 64 ears (36%) passed in the third round with AABR (Table 2).

**Table 2:** Characteristics per ear

		N_ears_ = 205 N (%)	N_OAE_ = 115 N (%)	N_AABR_ = 64 N (%)	*P*-value
Test pass NHS	OAE1 OAE2 AABR Missing data	105 (58.7) 10 (5.6) 64 (35.7) 26			
Type HL	Sensorineural Mixed	158 (77.0) 47 (23.0)			
Severity HL	Mild Moderate Severe Profound	49 (23.9) 95 (46.3) 42 (20.5) 19 (9.3)	33 (28.7) 45 (39.1) 21 (18.3) 16 (13.9)	13 (20.3) 34 (53.1) 15 (23.4) 2 (3.1)	0.906
Progression HL	Yes No Missing data	25 (83.3) 5 (16.7) 175			
Audiogram configuration	Low-frequency Mid-frequency High-frequency gradual High-frequency steep Flat Missing data	41 (20.1) 29 (14.2) 28 (13.7) 41 (20.1) 65 (31.9) 1	26 (22.6) 9 (7.8) 18 (15.7) 23 (20.0) 39 (33.9)	7 (11.1) 16 (25.4) 8 (12.7) 16 (25.4) 16 (25.4) 1	0.009

### Hearing loss

Evaluation of audiometry at presentation showed that 101 patients (85%) presented with bilateral HL, of which 66% was symmetrical (Table 1). Among the 205 ears assessed, SNHL was present in 158 ears (77%), while mixed HL was observed in the remaining 47 ears (23%) (Table 2). The severity of HL was classified as mild in 49 ears (24%), moderate in 95 ears (46%), severe in 42 ears (21%), and profound in 19 ears (9%). No significant differences were found when comparing the severity across different age groups, nor the type of NHS screening (i.e. OAE or AABR).

Ten years audiometry was available for eight out of 205 ears. Of these eight ears, five ears did not show progression of HL and three did (37%). Additionally, progression (≥ 15 dB increase in HL threshold) could be determined in 22 out of 197 ears within the ten-year period.

The most frequently observed audiogram configuration was flat (32% of the ears), followed by high-frequency steep and low-frequency HL (each 20% of the ears) and mid-frequency and high-frequency gradual HL (each 14% of the ears). A significant difference in audiogram configuration was observed between ears that passed via OAE and AABR (p = 0.009). Low-frequency HL was more common in the OAE group (23%) than in the AABR group (11%), while mid-frequency HL was more frequent in the AABR group (25%) compared to the OAE group (8%) (Table 2). In the logistic regression model audiogram configuration was also significant (p = 0.032), with children showing a mid-frequency loss being more likely to pass only at AABR (OR = 3.6, 95% CI 1.0–13.6, *p* = 0.049).

### Etiological diagnostics of HL

**Fig. 2:**
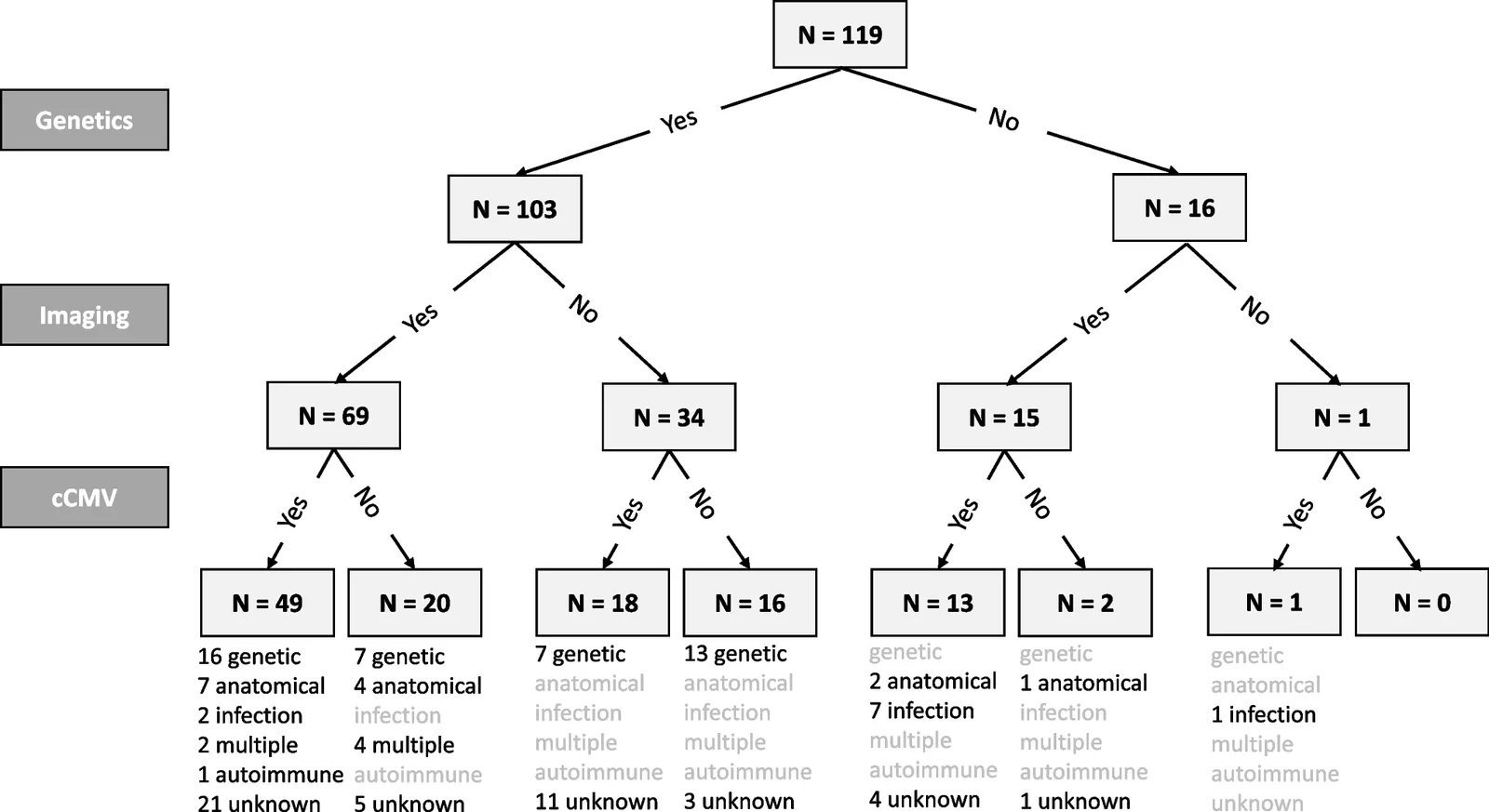
Diagnostic evaluation tree with diagnoses found (*N* = number of patients)

A comprehensive etiological work-up was performed in most patients: genetic testing in 103 patients (87%), imaging in 84 patients (71%) and cCMV testing in 81 patients (68%) (Fig. 2). A total of 49 patients (41%) underwent all three diagnostic evaluations. Twenty-four patients with an unknown cause did not undergo a complete diagnostic work-up. In most of these patients, a plausible explanation for omitting further diagnostics was identified. For example, four out of five patients who did not receive genetic testing had unilateral HL, which is not eligible for genetic testing in our center as genetic testing rarely leads to a diagnosis for unilateral HL. Additionally, two out of fourteen patients did not receive imaging due to age-related restrictions and newborn blood spot test samples were unavailable in four out of ten patients who did not receive cCMV analysis. In the other cases, further diagnostic testing was not performed due to a lack of parental consent.

### Etiology

**Fig. 3:**
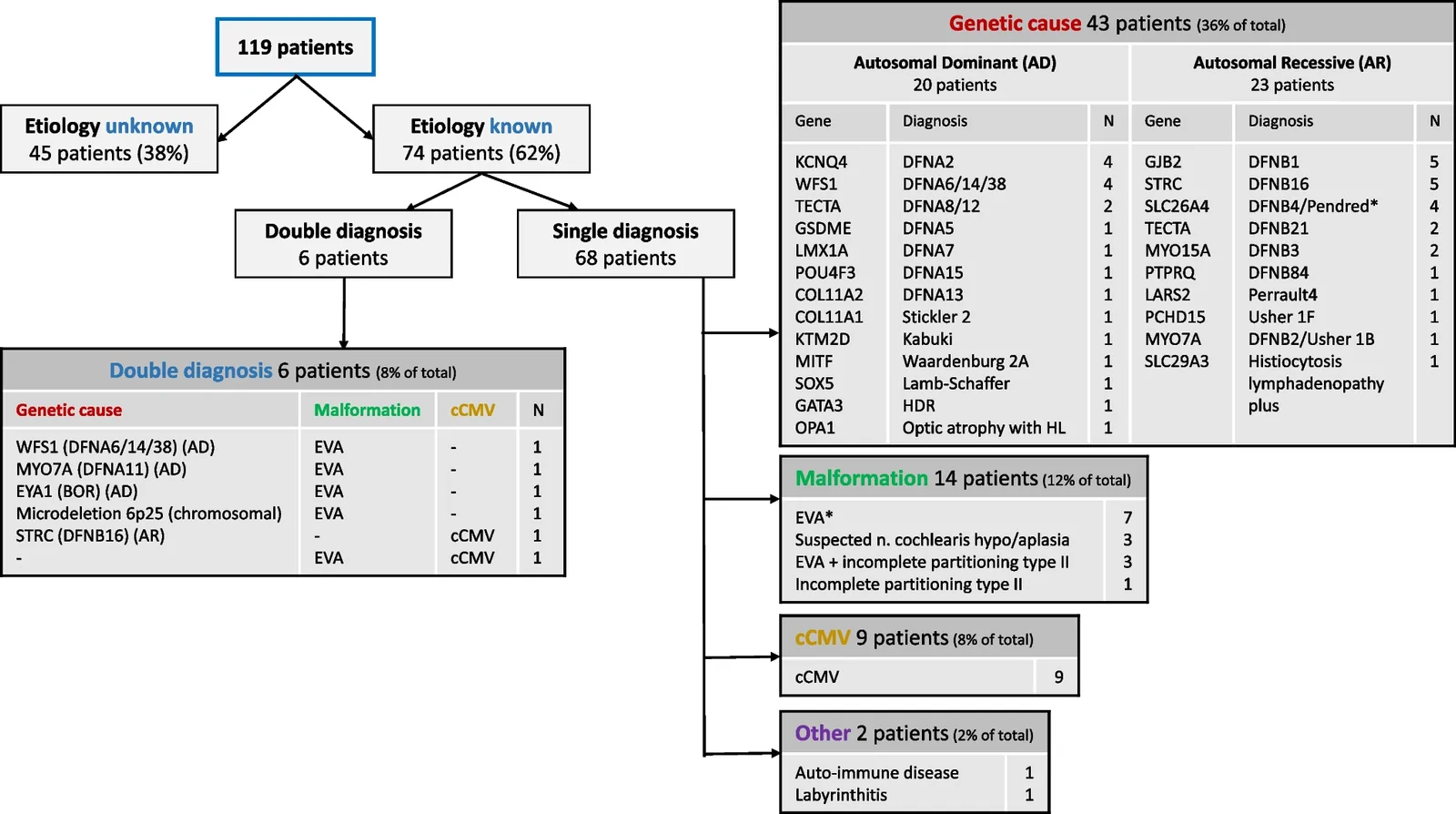
Etiology

A cause of HL was identified in 74 patients (62%), of which 6 patients had a double diagnosis (Fig. 3). In total, 48 patients were found to have a genetic cause of SNHL. A large variety of genetic diagnoses was found. Genetic diagnoses showed an equal distribution of autosomal dominant (AD) and autosomal recessive (AR) inheritance patterns. The most frequently identified genetic disorders were DFNB16 (*STRC)* in six patients, DFNB1 *(GJB2*) and DFNA6/14/38 (*WFS1*), both in five patients. In 16 patients, the identified genetic variant was associated with a (possibly) syndromic disorder.

Cochleovestibular malformations associated with SNHL were found in 22 patients. The most common malformation was an enlarged vestibular aqueduct (EVA), observed in 19 patients. Additionally, three patients showed cochleovestibular malformations on CT suggestive of cochlear nerve hypo/aplasia. Unfortunately, an MRI was not performed to confirm these findings, likely due to age-related limitations. In 17 patients, cochleovestibular malformations were located on the side(s) corresponding to HL. In two patients with bilateral HL, the cochleovestibular malformation was found unilaterally. However, one of these patients was additionally diagnosed with a genetic condition.

In 11 patients cCMV infection was identified as an underlying cause of HL, of which 2 had a double diagnosis. Both autoimmune inner ear disease and labyrinthitis were documented to be the most likely cause of HL in one patient.

The etiology of HL was not found in 45 patients (38%) after diagnostic evaluation. Notably, no patients with HL caused by auditive neuropathy spectrum disorders (ANSD) were present in this study. When excluding ears with mild SNHL, as NHS likely does not detect these losses, a cause of HL was identified in 58% of patients and the distribution between groups of etiology did not change significantly.

### Additional clinical data

#### Speech and language development (SLD)

**Fig. 4:**
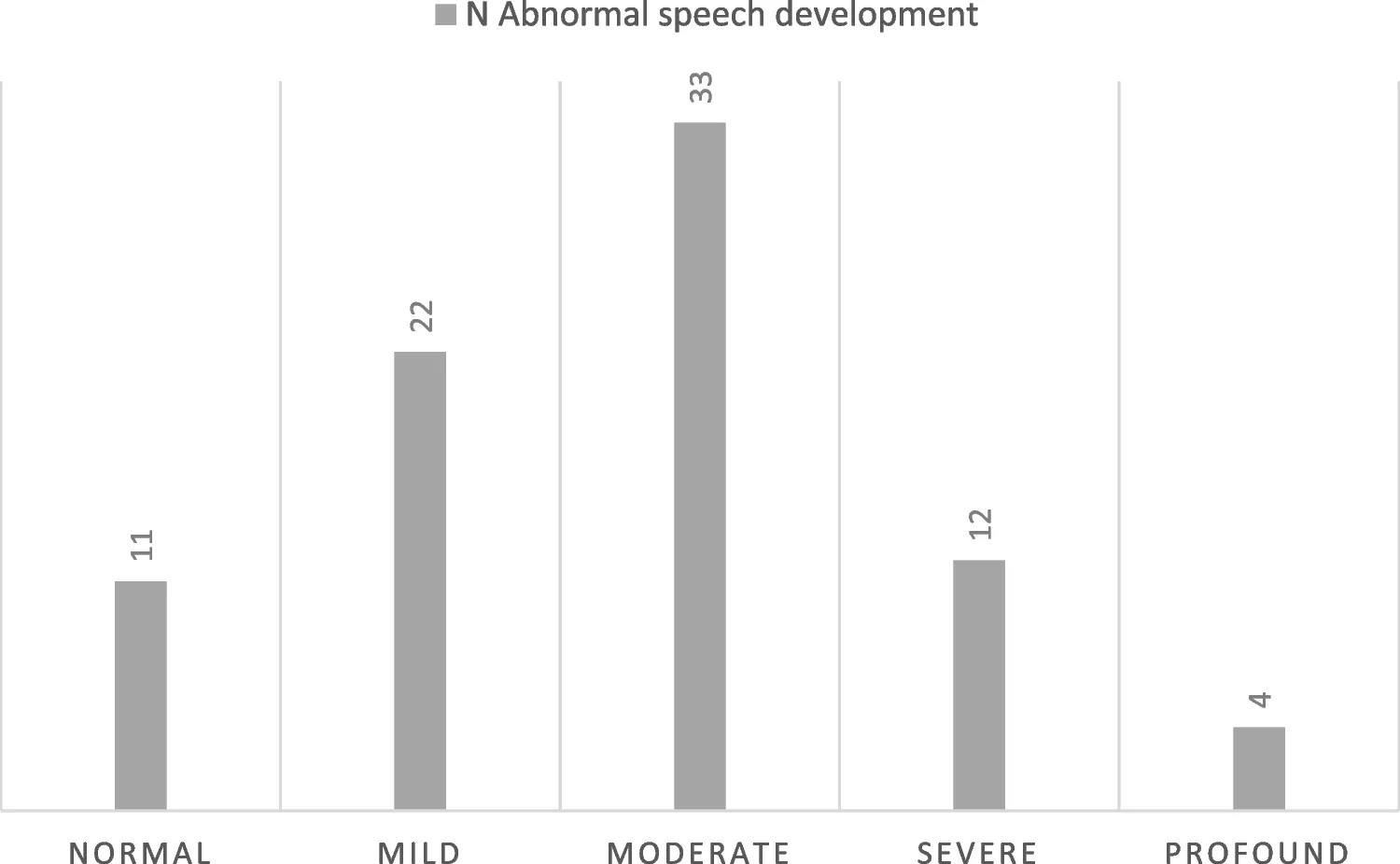
Severity of HL in best hearing ear in patients with abnormal SLD (*N* = 82)

In 10 patients no data on SLD was present. An abnormal SLD was reported in 82 of the 109 patients (75%). Among these patients 90% showed delayed SLD, 7% stagnated SLD and 2% no development of speech and language (Table 1). Given that SLD is primarily influenced by the hearing status of the best hearing ear, it is notable that abnormal SLD occurred across all levels of HL in the best hearing ear (Fig. 4), including 11/82 patients with unilateral normal hearing. Among these 11 patients, eight had severe or profound HL in the affected ear and three had mild-moderate HL. Interestingly, cCMV was the most frequently identified etiology in this subgroup, observed in seven out of eleven patients (64%), suggesting that broader neurodevelopmental factors may contribute to the observed SLD.

#### Family history

**Fig. 5:**
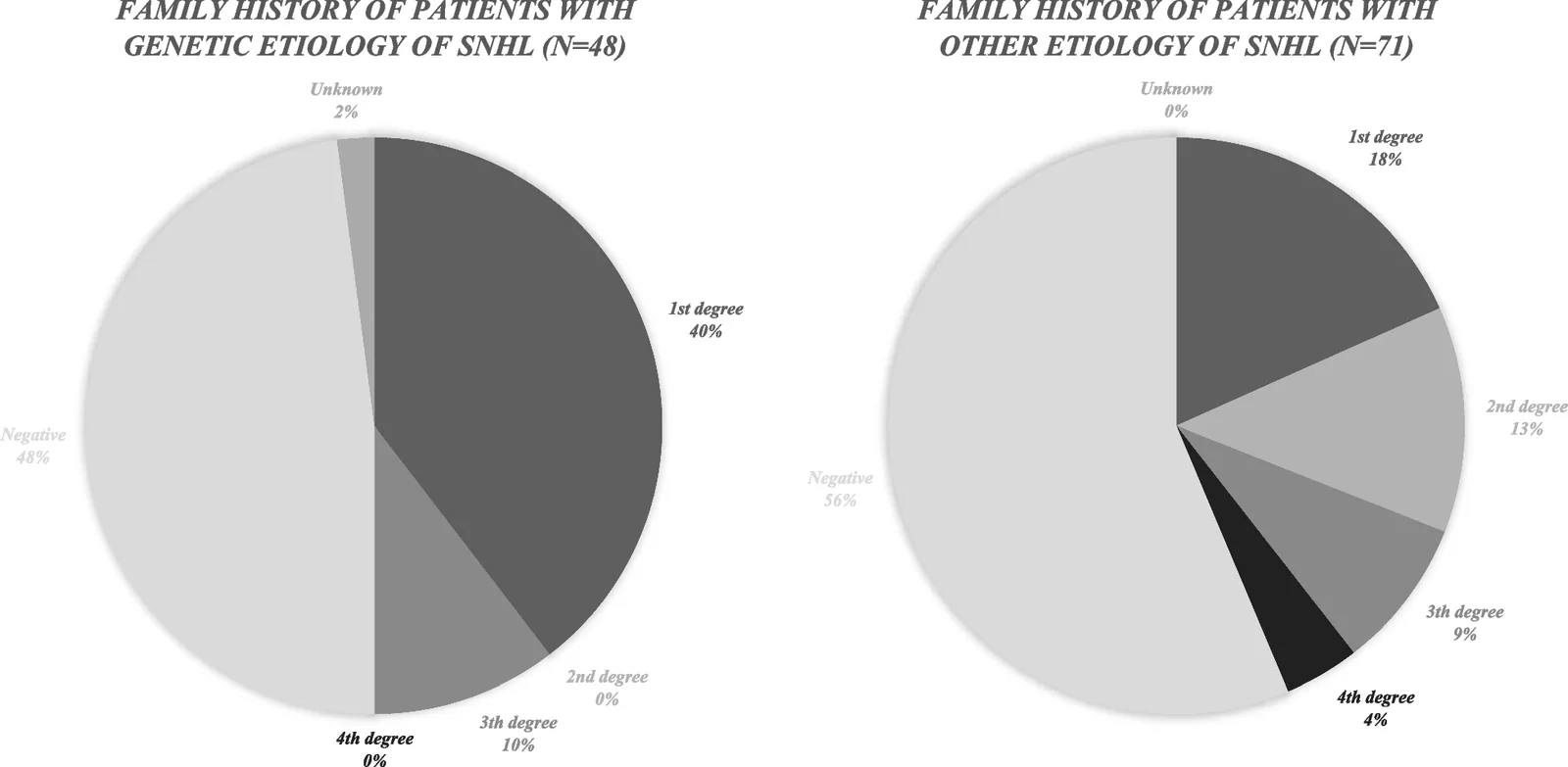
Family history

A positive family history of SNHL was reported in 55 patients (46%). Within the genetically diagnosed group, a positive family history in a first-degree relative was reported more than twice as often compared to the group without a genetic diagnosis; 40% vs. 19%, respectively (Fig. 5).

#### Risk factors

Known risk factors for SNHL include asphyxia, hyperbilirubinemia, recurrent otitis, ototoxic medication, meningitis, head trauma, admittance to NICU, and noise exposure. Because only patients with an unknown cause of HL were included in this study, documented risk factors where not the leading cause of HL in our patients. Nevertheless, perinatal asphyxia was documented in three patients (3%) and nine patients (8%) presented with postnatal hyperbilirubinemia, with one patient requiring treatment. Recurrent otitis was reported in 37 patients (31%). Exposure to possible ototoxic medication during pregnancy or childhood was identified in four cases (3%), involving gentamicin (*n* = 2), amoxicillin (*n* = 1) and lamotrigine (*n* = 1). One patient had a history of meningitis, however, the hearing loss was present prior to this infection. Head trauma was reported in six patients (5%), three of whom had cochleovestibular malformations, including one case of EVA, one case of incomplete partitioning and one case with a combination of both. Eleven patients (9%) were admitted to the NICU shortly after birth. There was no report of noise-induced HL. No significant differences were found in the prevalence of risk factors across categories of etiology.

## Discussion

The Dutch NHS program successfully identifies approximately 200 patients with HL each year, facilitating early intervention and thereby improving developmental outcomes [7]. Nevertheless, a substantial number of patients are still diagnosed with SNHL below the age of five, despite initially passing the NHS. This subgroup is heterogeneous in terms of HL characteristics and underlying etiologies. Notably, abnormal SLD was present in 75% of these patients, underscoring the need for earlier detection strategies. However, given the diversity within this group, this poses a significant challenge.

### A substantial number of patients

Over an 11-year period, 119 patients under five years of age were identified with SNHL that passed the NHS. Of these, 105 patients (approximately 11 per year) passed the NHS bilaterally and were therefore not referred for further diagnostic hearing workup. Based on the assumption that our center receives approximately 20% of such cases nationwide, this suggests that about 50 patients per year may present with SNHL before the age of five despite having passed NHS in the Netherlands. This likely underestimates the true prevalence, as it includes only those who are referred for etiological evaluation of their HL. Although lower than the roughly 200 cases detected through NHS each year, this represents a substantial number of potentially later diagnosed patients. These findings underscore the importance of optimizing early HL detection programs and highlight the need for targeted improvements to reduce the number of missed or delayed diagnoses.

### Heterogeneous characteristics

The study population comprised a heterogeneous group concerning the severity of HL, audiogram configuration and etiological cause. An etiological cause was established in 62% of patients. This diagnostic yield is consistent with previous studies reporting that the etiology of SNHL remains unknown in 25–45% of patients after diagnostic workup [16].

The etiology of hearing loss was attributed to genetic disorders in 40% of patients (including patients with multiple diagnoses), consistent with previous studies reporting that approximately 50% of congenital SNHL is attributed to genetic causes [1, 16]. In contrast, Dedhia et al. reported a lower proportion (17%) of genetically confirmed cases among patients with SNHL who had passed their NHS [13]. The higher prevalence of genetic diagnosis observed in our study may be attributable to the larger portion of patients that underwent genetic testing (87% versus 50%) and the more extensive type of sequencing applied in our center (whole exome sequencing versus targeted sequencing of *GJB2/6* and *SLC26A4* only). Approximately half of the genetic disorders identified in this study had an autosomal dominant inheritance pattern, which is substantially higher than the typically reported 15–20% in genetic SNHL [1, 17] This discrepancy may be due to the fact that a positive family history occurs more frequently in autosomal dominant inherited HL, making parents more aware and motivated to seek further HL testing for their children. Additionally, in general, autosomal dominant inherited HL is more often post-lingual, mild and progressive, while autosomal recessive HL is more likely prelingual and severe or profound [18]. Thus, cases of autosomal recessive inherited HL are more likely to be detected through NHS program and may be underrepresented in this cohort.

Regarding other etiologies, cochleovestibular malformations, predominantly EVA, were identified as the cause of HL in 18% of patients. This finding aligns with previous research indicating that approximately 20% of children with SNHL present with inner ear malformations, and with the 14% prevalence of structural abnormalities reported by Dedhia et al. among patients who passed NHS [13, 19]. Congenital CMV infection accounted for SNHL in 9% of patients, consistent with earlier studies estimating that cCMV causes 10–20% of SNHL in children [20]. Although it is reported that 7–10% of all childhood HL is due to ANSD, it is notable that in our study no patients with ANSD were present [21]. This absence may be attributed to the study protocol of the NHS, where the first two rounds consist of OAE screening. Patients with ANSD can display normal OAE results, since OAE’s primarily reflect outer hair cell function, and they will receive no further screening if they pass the first or second screening round [1]. In our study infants were not included when they followed the alternative NICU protocol, consisting of two rounds of AABR screening. The NICU screening protocol is designed for newborns admitted to the NICU for > 48 h, because they have a significant higher chance of SNHL and ANSD.

It should be noted that patients with a known etiology of SNHL prior to presentation were excluded in this study, which likely resulted in an underrepresentation of children with a known cCMV infection at birth and common acquired etiologies such as meningitis, otitis or ototoxic drug exposure.

Furthermore, we investigated the differences in characteristics between ears that passed with OAE and AABR testing in this group. Analysis of audiograms revealed a significant difference between the two screening methods. Specifically, patients with low-frequency SNHL passed significantly more frequently with OAE screening. This finding may be attributed to the narrower frequency sensitivity of OAE testing (1000-4000 Hz) compared to AABR (750-5000 Hz) [10, 11]. Conversely, patients with mid-frequency HL passed significantly more with AABR screening. Given that both low- and high-frequencies, which are typically unaffected in mid-frequency HL, are encompassed in the AABR’s broader frequency range, these may generate a sufficient response in AABR testing, whereas OAE may not detect such responses with its narrower range.

Based on clinical observations, we hypothesized that a substantial proportion of patients in our cohort passed NHS only in the third round, using AABR. As shown in Fig. 1, most newborns pass the NHS in the first round with OAE, while only a small number (approximately 2,300 of the 167,000 newborns screened) pass in the third round with AABR. In contrast, in this study 36% of ears passed in the AABR stage, this is a disproportionately high percentage compared to the general screening population.

### Reasons for passing NHS

Our findings prompted further investigation into why these specific cases were not detected during NHS. Several possible explanations may account for the presence of SNHL after passing NHS.

First, mild HL can remain undetected during NHS, as screening methods are designed to identify HL greater than 35–40 dB. In our study, 24% of ears presented with mild HL. Similarly, Dedhia et al. found that 32% of patients with SNHL after passing NHS presented with mild HL [13].

Second, false negatives could potentially have occurred. Certain etiologies are known to be at risk for false-negative outcomes during NHS. For instance, ANSD and cochlear nerve dysplasia can be missed due to normal OAE results [1, 22, 23]. However, in our study population all three patients diagnosed with possible cochlear nerve dysplasia were referred after OAE screening, but passed AABR screening afterwards. Although in all these three patients the hypoplasia was suggested on CT, but not confirmed with MRI, we cannot completely elucidate these findings. These might be examples of true false negative NHS results. On the other hand varying hearing thresholds in cochlear nerve hypoplasia have been described [24]. Thus, while the occurrence of false negatives in NHS can not be entirely ruled out, this is likely rare, and our findings did not provide definitive evidence supporting the opposite. Additionally, it is reported that approximately 10% of patients with DFNB1-related SNHL are not detected with OAE screening [25]. This is likely due to the variable severity of HL in DFNB1 (mild to profound), whereas mild HL is not detected with the screening thresholds (> 40 dB). However, in our study all five patients with DFNB1 were referred after OAE screening, but passed AABR screening afterwards.

Finally, early-onset or progressive SNHL may not be detected by NHS, as SNHL is not present or mild during the time of the screening. SNHL caused by cCMV can be both late-onset and progressive [20, 26, 27]. Fowler et al. reported that 43% of infants with cCMV-related SNHL were not identified through NHS [28]. Similarly, SNHL associated with EVA may present as early-onset, progressive or fluctuating [29, 30]. A retrospective study by Perry et al. found that 46% of patients with confirmed SNHL caused by EVA passed NHS [31]. Genetic SNHL, especially autosomal dominant inherited SNHL, can also manifest after the neonatal period or progress over time, and may therefore be missed by NHS [17, 18]. Although data on the progression of HL in our study was limited, progression was still present several ears.

### Possibilities for early detection

As previously established, delayed detection of HL can negatively impact SLD. In this study, approximately three-fourth of patients had an abnormal SLD, underscoring the urgent need for earlier identification. The question then arises: how can earlier detection be achieved?

Analysis of the clinical characteristics and reasons for passing NHS provides valuable insight into the potential methods for earlier detection. However, missed cases due to mild HL or false negatives are unlikely to be fully resolved through modifications of the current screening protocol. Analysis of patient characteristics revealed a highly heterogeneous group, with genetic causes forming the largest subgroup. Yet no single genetic etiology predominated, suggesting that targeted genetic testing is not currently feasible or sufficient.

Notably, a positive family history of SNHL in a first-degree relative was significantly more common in patients with a genetic etiology compared to those with other etiologies (40% versus 19%, respectively). This may be attributable to the relatively high proportion of autosomal dominant inheritance observed in our study. Nevertheless, referral of children with a positive family history of SNHL in a first-degree relative for further audiologic or genetic evaluation may be warranted, as these infants appear to be at increased risk. Such risk-based assessments could feasibly be integrated into routine pediatric care as part of a broader early identification strategy.

In addition to genetic factors, cCMV infection poses a significant risk for SNHL with late diagnosis. Notably, seven out of eleven patients with unilateral HL and an abnormal SLD were diagnosed with cCMV. Early identification of cCMV-infected infants may enable treatment, close audiological monitoring and timely intervention upon onset of SNHL. A recent consensus statement on the management of cCMV advocates for maternal CMV serology screening during the first trimester of pregnancy to facilitate this [32].

Furthermore, a significant difference was found between OAE and AABR in the audiogram configurations. However, as both tests have a risk of missing HL with certain audiogram configurations because of differing frequency ranges, we do not recommend modifications to the current screening protocol based on this finding. Nevertheless, further research comparing the diagnostic effectiveness of OAE versus AABR may provide valuable insights.

Finally, given that early-onset or progressive HL may be missed in the NHS, the implementation of a second hearing screening before the age of three could facilitate earlier detection. While early comprehensive diagnostic evaluation and rehabilitation of HL before the age of six months is standard clinical practice and is proven to be the most beneficial for SLD, social development, as well as overall quality of life, current evidence also supports these benefits even when initiated after six months of age. For instance, studies on cochlear implantation indicate that implantation before 36 months is associated with superior developmental outcomes compared to later intervention [1, 33]. Additionally, a study by Ching et al. on 5-year language outcomes showed that the younger the interventions are implemented, the better the language outcome [4]. Considering this evidence, screening at about two years of age could be particularly beneficial, allowing for timely intervention before the age of three. In our study, 73% of children were referred after the age of three for etiological diagnostics, although most had already been diagnosed with SNHL before referral. We hypothesize that, besides improving parental awareness of early signs of HL and developmental delay, a second screening conducted at two years of age could identify a substantial number of patients with late-onset or progressive SNHL and maximizes the potential for improved SLD outcomes. However, re-screening all children in the Netherlands may not be feasible and cost-effective. Therefore, a targeted follow-up screening could be a pragmatic alternative. Subgroups that should be considered for additional screening are children who passed the NHS in the third screening round with AABR or children who have a positive family history in a first-degree relative, as they represented a relatively large percentage in this cohort, while the number of patients to re-screen is low. Additionally, children with a developmental delay and parental concerns should be referred for complete audiologic evaluation. Prospective studies are required to quantify the number of patients with SNHL that can be identified through this approach and to evaluate the clinical efficacy and cost-effectiveness.

### Limitations

Our study has some limitations inherent to its retrospective descriptive design. Complete data could not be obtained for certain variables. In addition, some diagnoses may be missed, as not all patients underwent all available diagnostic evaluations.

## Conclusion

Based on our findings, annually approximately 50 patients present with SNHL before the age of five each year after having passed NHS in the Netherlands. This is a substantial number of patients, who are at risk for delayed detection and rehabilitation, which causes poorer developmental outcomes. Achieving earlier diagnosis may be challenging due to the diversity within this group, especially regarding the severity and audiogram configuration of the HL and its etiology. Future research is warranted to investigate the genotype–phenotype correlations associated with genetic diagnoses within this cohort, thereby enhancing the understanding of why these patients present with SNHL after passing NHS. Possibilities for earlier detection of these children include conducting cCMV testing via maternal serology, and implementing a second screening at the age of two for those who passed the NHS in the third round with AABR and for those with a positive family history for SNHL. Furthermore, prospective studies are essential to evaluate the effectiveness of proposed implementations for earlier detection and its clinical consequences regarding SLD.

## Abbreviations

NHS: Newborn hearing screening
OAE: Otoacoustic emissions
AABR: Automated auditory brainstem response
HL: Hearing loss
SNHL: Sensorineural hearing loss
SLD: Speech and language development
NICU: Neonatal intensive care unit
PTA: Pure tone average
CT: Computed tomography
MRI: Magnetic resonance imaging
cCMV: Congenital cytomegalovirus infection
AD: Autosomal dominant
AR: Autosomal recessive
EVA: Enlarged vestibular aqueduct
ANSD: Auditive neuropathy spectrum disorders
